# Prevalence and Genotype Distribution of Human Papillomavirus in Primary Lung Cancer in Eastern India

**DOI:** 10.7759/cureus.115322

**Published:** 2026-08-27

**Authors:** Isha K Khobragade, Baijayantimala Mishra, Pritinanda Mishra, Prasanta R Mohapatra, Srujana Mohanty

**Affiliations:** 1 Microbiology, All India Institute of Medical Sciences, Bhubaneswar, Bhubaneswar, IND; 2 Pathology, All India Institute of Medical Sciences, Bhubaneswar, Bhubaneswar, IND; 3 Pulmonary Medicine and Critical Care, All India Institute of Medical Sciences, Bhubaneswar, Bhubaneswar, IND

**Keywords:** adenocarcinoma, ffpe tissue, hpv-16, hpv-18, human papillomavirus, lung cancer, non-small cell lung cancer, real-time pcr, smoking, squamous cell carcinoma

## Abstract

Background: Lung cancer is the leading cause of cancer-associated mortality globally, with approximately 2.5 million new cases and 1.8 million deaths. It accounts for approximately one in five cancer-related deaths worldwide. While tobacco smoking is the main cause of lung cancer, many cases occur in never-smokers, indicating that other environmental, genetic, and infectious contributors may play a role. High-risk human papillomavirus (HPV), particularly HPV-16 and -18, is a known etiologic agent in cervical, anogenital, and oropharyngeal malignancies. The pathogenic role of HPV in lung cancer remains controversial, mainly due to significant geographical variation in prevalence and inconsistent detection rates, despite increasing molecular evidence of HPV DNA in primary lung tumors. Information about HPV-related lung cancer in India is scarce. Objectives: This study aims to determine the prevalence of high-risk HPV types 16 and 18 in primary lung cancer tissue samples and to evaluate their potential associations with clinicodemographic characteristics. Materials and methods: A retrospective cross-sectional study was conducted using samples collected between January 2023 and October 2025 at a tertiary care teaching hospital in eastern India. A total of 95 archival formalin-fixed paraffin-embedded (FFPE) tissue specimens from histopathologically confirmed primary lung neoplasms were included in the study. DNA was extracted from FFPE tissues and subjected to real-time polymerase chain reaction for HPV-16 and HPV-18. We compared clinicodemographic variables between HPV-positive and HPV-negative patients. Results: HPV-16 DNA was detected in 29 of 95 (30.5%) lung cancer specimens, whereas HPV-18 was not detected in any case. Squamous cell carcinoma showed a higher HPV-16 positivity rate than adenocarcinoma (42.1% vs. 26.8%), although the difference was not statistically significant. HPV-positive patients had a mean age of 64 years and were predominantly male. Smoking was significantly associated with HPV positivity (p = 0.035). Most HPV-positive tumors involved the right lung and occurred among rural residents. Conclusions: Of primary lung cancers in this cohort, one third were HPV-16 positive, and none were HPV-18 positive. These findings suggest a possible association between HPV-16 infection and pulmonary carcinoma, particularly among smokers. Future larger multicenter studies may clarify the biological relevance of HPV in lung carcinogenesis by incorporating detailed viral oncogene expression and genotyping.

## Introduction

Lung cancer is the second most commonly diagnosed malignancy. It continues to be the leading cause of cancer-related death worldwide, accounting for nearly one in every five cancer deaths [1,2]. The five-year survival remains dismal because most patients present with advanced disease [2], despite remarkable advances in imaging, molecular diagnostics, targeted therapy, and immunotherapy. Although tobacco smoking is still the main etiological factor, about 10-25% of lung cancers develop in individuals with little or no history of smoking, suggesting that other environmental, occupational, genetic, and/or infectious factors may enhance pulmonary carcinogenesis [3,4].

Human papillomavirus (HPV) is a small, double-stranded, non-enveloped DNA virus from the Papillomaviridae family. Over 220 different HPV genotypes have been characterized to date, with HPV-16 and HPV-18 contributing to almost 70% of cervical cancers, as well as a range of noncervical malignancies, such as anogenital (vulva, vagina, penis, and anus) and oropharyngeal cancers [5]. Infection with these high-risk HPV types of HPV leads to malignant transformation due to expression of the viral oncoproteins E6 and E7, which bind to and inactivate the tumor suppressor proteins p53 and retinoblastoma, causing loss of contact inhibition, self-sufficient cell replication, insensitivity to growth inhibitory signals, evasion of apoptosis, chromosomal instability, and accumulation of mutations during cellular proliferation [5,6].

The role of HPV in lung carcinogenesis has been debated. This association was first proposed by Syrjänen in 1979 after demonstrating HPV-associated cytopathic changes within the bronchial epithelium [7]. Following this observation, HPV DNA has been studied in primary lung tumors, and the prevalence has been found to range from 0% to more than 60%, reflecting high variability in study design, geographical distribution, molecular detection methods, and sample selection [8,9]. A recent systematic review and meta-analysis estimated an overall HPV prevalence of ~13.5% in more than 9,000 lung cancer patients, with higher rates detected in Asia compared to Europe or North America [10]. In most of these studies, HPV-16 has been consistently observed as the predominant type, with less frequent detection of HPV-18 [10,11]. The varied geographical distribution of HPV genotypes highlights the need for more studies to clarify the prevalence and clinical impact of HPV in lung cancer in various populations.

In India, most of the pivotal research conducted on HPV-related cancers has focused only on cervical cancer, and data regarding the HPV infection status in lung cancer is still very sparse. This study was conducted to assess the prevalence of high-risk HPV genotypes (HPV-16 and HPV-18) in patients with histopathologically proven primary lung cancer using real-time polymerase chain reaction (PCR) at a tertiary care center in eastern India and to examine the association between HPV positivity and various clinicodemographic variables.

## Materials and methods

This retrospective cross-sectional observational study was conducted on samples between January 2023 and October 2025 in the Departments of Microbiology, Pulmonary Medicine, and Pathology at the All India Institute of Medical Sciences (AIIMS), Bhubaneswar, India, which started on January 21, 2025, in accordance with the principles of the Declaration of Helsinki [12]. The HPV DNA testing was not done at the time of diagnosis of lung cancer; rather, all the samples collected over three years were tested later. Ethical approval was obtained from the Institutional Ethics Committee of AIIMS, Bhubaneswar (approval number: IEC/AIIMS BBSR/PG Thesis/2023-24/153).

Study population

Formalin-fixed paraffin-embedded (FFPE) tissue samples obtained from patients who were diagnosed with primary lung carcinoma by histopathology were included. An experienced pulmonary pathologist selected representative tumor-rich regions within each FFPE tissue block. Exclusion criteria were metastatic lung malignancies, pulmonary sarcomas, recurrent tumors, or tissue blocks with insufficient material for DNA extraction.

Sample size calculation

The required sample size was calculated taking an approximate frequency of 25% using the following equation:

$n = \frac{z_{\alpha}^{2} p q}{d^{2}}$

where p is the expected proportion of HPV-16 and HPV-18 in the cases (6.3), q is the expected proportion of other types of HPV (93.7), and d is the desired level of precision (5), which gave a sample size (n) of 94. The minimum required sample size was calculated to be 94; consequently, a total of 95 eligible cases were enrolled.

DNA extraction and HPV detection

**Figure 1:**
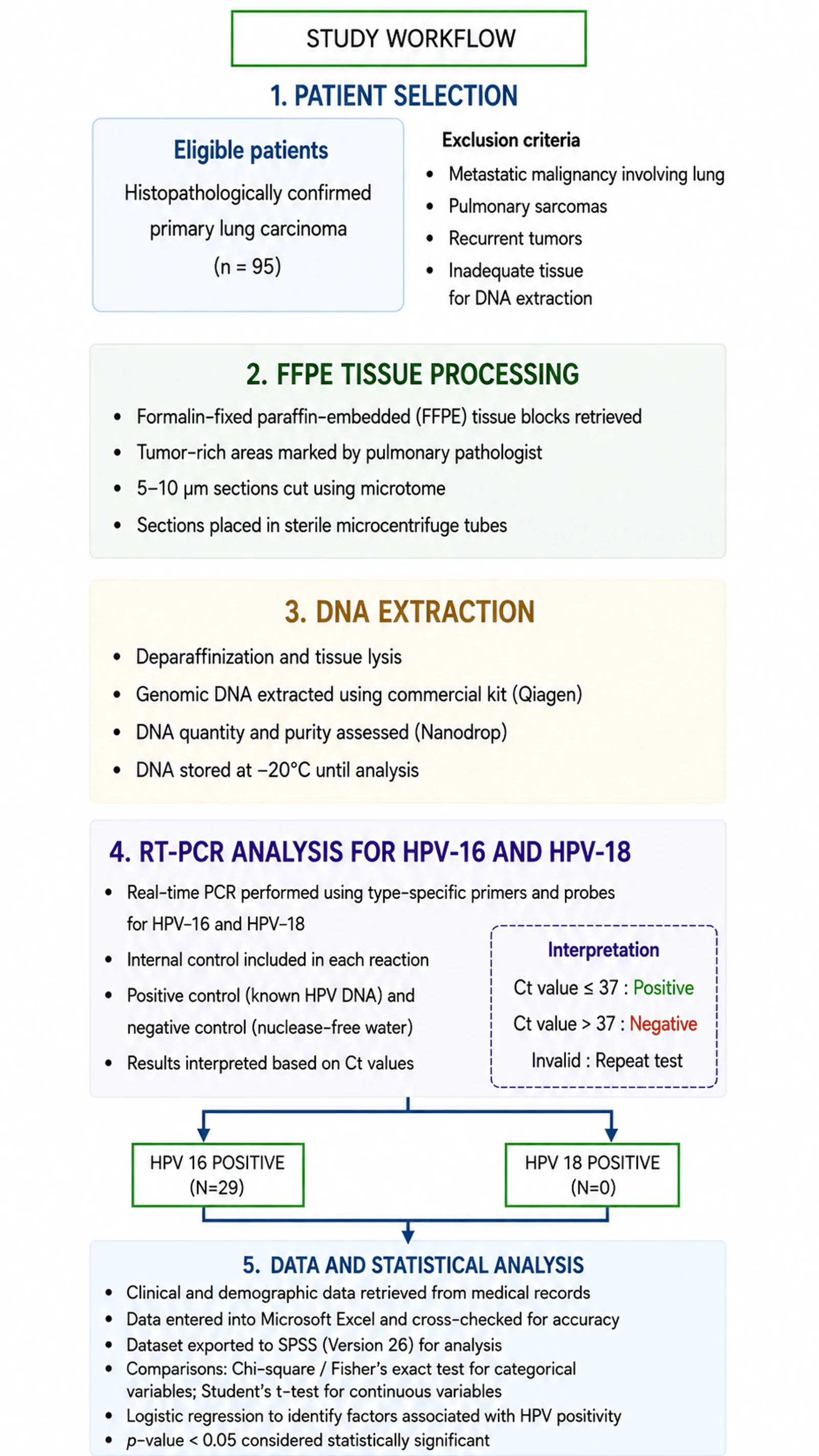
Flow diagram showing patient selection, FFPE tissue processing, DNA extraction, real-time PCR analysis for HPV-16 and HPV-18, and final statistical analysis FFPE: formalin-fixed paraffin-embedded, DNA: deoxyribonucleic acid, PCR: polymerase chain reaction, HPV: human papillomavirus

Genomic DNA was extracted using the QIAamp DNA FFPE Tissue Kit (QIAGEN, Hilden, Germany) according to the manufacturer's instructions. DNA quantity and quality were assessed using a NanoDrop spectrophotometer (Thermo Fisher Scientific Inc., Waltham, MA, USA) by measuring absorbance at 260 and 280 nm. Detection of high-risk HPV genotypes 16 and 18 by real-time PCR using commercially available primers and probes (TRUPCR HPV-16 and HPV-18 detection kit, 3B BlackBio Dx Ltd, India) was performed according to the manufacturer's instructions along with appropriate positive and no-template controls as provided in the kit. The study workflow is shown in Figure 1.

Data collection and statistical analysis

Clinical and demographic data were obtained from hospital medical records, including age, sex, smoking history, passive smoking, area of residence, histological subtype, side of the involved lung, and survival status. Histological diagnosis and subtype evaluation of lung cancer were performed in accordance with the current World Health Organization classification of thoracic tumors. Data were organized in Excel (Microsoft Corp., Redmond, WA, USA) and analyzed using SPSS Statistics version 26.0 (IBM Corp., Released 2019; IBM SPSS Statistics for Windows, Version 26.0; Armonk, NY, USA). Depending on whether the data were normally distributed, the quantitative results were reported as the mean and standard deviation or as the median with the interquartile range. The proportion and confidence interval were used to display the categorical data. The independent-samples Student's t-test was used for normally distributed continuous variables, while the categorical variables were compared using the Chi-square test or Fisher's exact test, as appropriate. Bivariate logistic regression was used to identify the risk factors associated with the development of high-risk HPV. In the multivariable analysis, the p-value threshold was set at <0.05 to indicate statistical significance.

## Results

A total of 95 patients with histopathologically confirmed primary lung carcinoma were included (Table 1). The mean age of the study population was 61.4 ± 11.4 years (age range 28-83 years). Most patients were male (69; 72.6%), yielding an overall male-to-female ratio of approximately 2.7:1. Rural residents accounted for 78.9% of the study population. Smoking history was present in 52.6% of patients, while passive smoking was reported by 13.7%. The right lung was more frequently involved (62.1%) than the left lung (31.5%). Bilateral disease occurred in only two patients (2.1%). At the end of the follow-up period, mortality was observed in 30.5% of patients.

**Table 1:** Baseline clinicodemographic characteristics of study participants

Characteristic		Number (%)
Age (years)	Range	28-83
	Mean	61.4 ± 11.4
	<55	22 (23.1)
	56-64	35 (36.8)
	65-75	26 (27.3)
	>76	12 (12.6)
Gender	Male	69 (72.6)
	Female	26 (27.4)
Type of residence	Rural	75 (78.9)
	Urban	20 (21.1)
Site of the lung involved	Right	59 (62.1)
	Left	30 (31.5)
	Bilateral	2 (2.1)
	Side not specified	4 (4.2)
Smoking status	Non-smoker	45 (47.4)
	Smoker	50 (52.6)
Outcome	Alive	66 (69.5)
	Dead	29 (30.5)

Histopathological distribution

Non-small cell lung carcinoma (NSCLC) accounted for the majority of tumors (90/95). Adenocarcinoma was the predominant subtype (71, 74.7%), followed by squamous cell carcinoma (19, 20.0%). Three cases were classified as NSCLC-not otherwise specified (NOS), while two patients had small cell lung carcinoma (SCLC). Among the 95 lung cancer specimens, 29 (30.5%) tested positive for HPV-16 DNA. None of the samples demonstrated HPV-18 DNA. Overall, HPV-positive tumors consisted predominantly of NSCLC (28/29; 96.6%).

HPV positivity according to histological subtype

**Figure 2:**
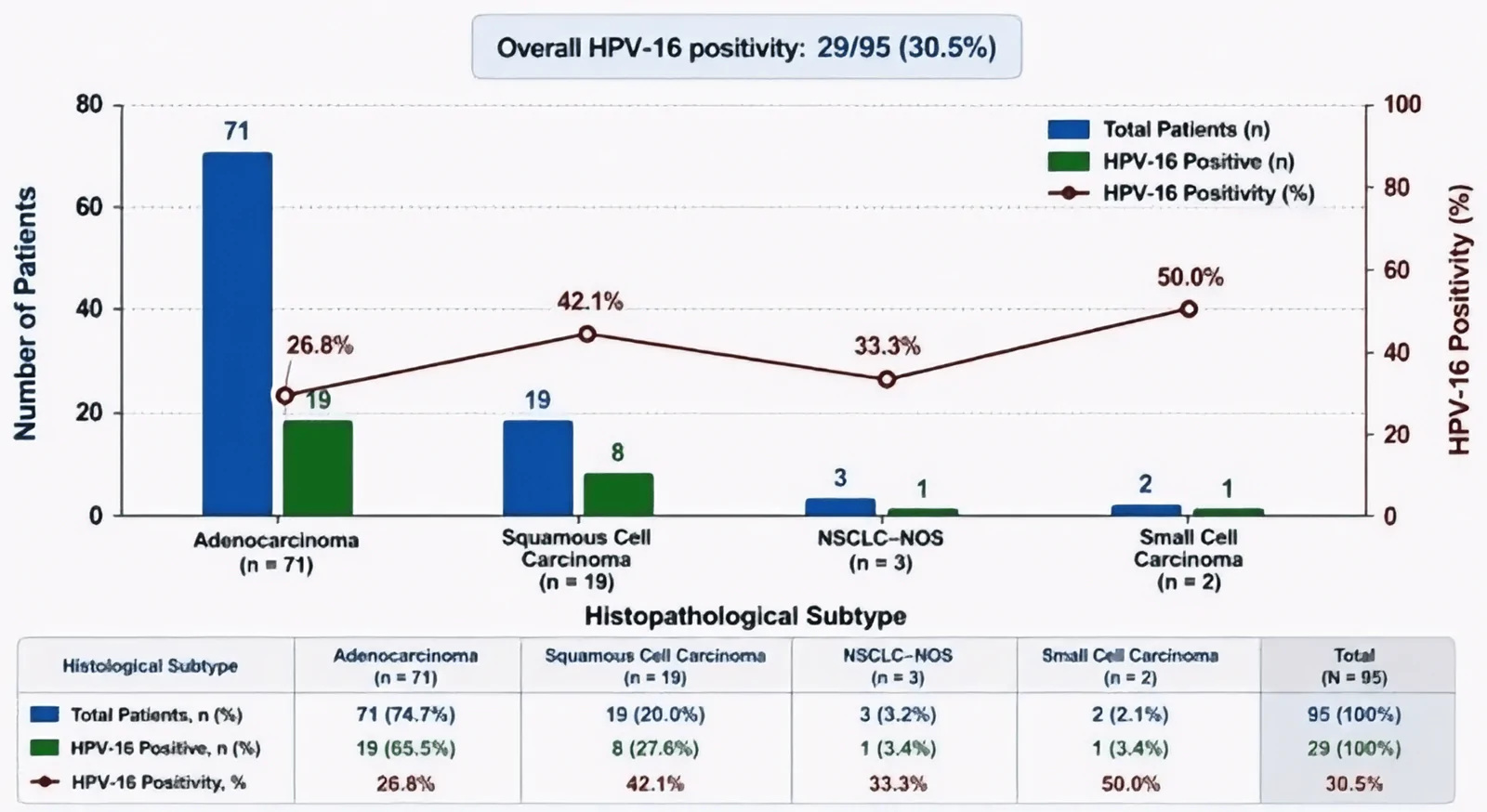
Distribution of HPV-16 positivity according to histopathological subtype of primary lung cancer The differences were not statistically significant (p > 0.0). HPV: human papillomavirus, NSCLC-NOS: non-small cell lung carcinoma-not otherwise specified

Although adenocarcinoma accounted for the largest number of HPV-positive tumors because it was the commonest histological subtype, the proportion of HPV positivity was higher in squamous cell carcinoma (Table 2, Figure 2). However, these differences were not statistically significant (p > 0.05).

**Table 2:** HPV-16 positivity according to histopathological subtype HPV: human papillomavirus, NSCLC-NOS: non-small cell lung carcinoma-not otherwise specified

Histology	Total (N)	HPV Positive (n)	HPV positivity (%)
Adenocarcinoma	71	19	19/71 (26.8)
Squamous cell carcinoma	19	8	8/19 (42.1)
NSCLC-NOS	3	1	1/3 (33.3)
Small cell carcinoma	2	1	1/2 (50.0)
Total	95	29	29/95 (30.5)

Clinicodemographic characteristics according to HPV status

A comparison of clinicodemographic characteristics of patients with HPV positivity to those of the HPV-negative population is detailed in Table 3, in which age was presented only descriptively, and the Chi-square test was used to assess the significance of categorical data. Patients with HPV-positive tumors had a mean age of 64 years, compared with 60 years among HPV-negative patients. Male predominance was observed in both groups. Smoking was significantly associated with HPV positivity. Nearly 69% of HPV-positive patients were smokers compared with 45% among HPV-negative patients (p = 0.035). Most HPV-positive patients were residents of rural areas (65.5%), while 35% of them were from urban areas. Right-sided lung involvement was observed in 69% of HPV-positive patients compared with 59% of HPV-negative patients. Mortality was similar between the two groups.

**Table 3:** Comparison between HPV-positive and HPV-negative patients ^#^Age is presented descriptively; ^*^p-values were calculated using the Chi-square test. HPV: human papillomavirus

Variable	HPV positive (N = 29)	HPV negative (N = 66)	p-value
Mean age (years)	64	60	-^#^
Male sex	21 (72.4%)	48 (72.7%)	0.98^*^
Smokers	20 (69.0%)	30 (45.5%)	0.035^*^
Rural residence	19 (65.5%)	56 (84.8%)	0.07^*^
Right lung involvement	20 (69.0%)	39 (59.1%)	0.31^*^
Mortality	9 (31.0%)	20 (30.3%)	0.94^*^

## Discussion

We conducted the current retrospective study to evaluate high-risk HPV infection in primary lung cancer using real-time PCR. We found that 30.5% of the retrieved specimens were positive for HPV-16, whereas HPV-18 was not detected in any specimen. HPV positivity was more frequent among smokers, patients with squamous cell carcinoma, and patients with right-sided tumors; however, only smoking was significantly associated with HPV positivity (p = 0.035). These results add to a growing body of evidence indicating that HPV-16 may be involved in pulmonary carcinogenesis among both smokers and nonsmokers, especially Asian patients.

In the past three decades, infectious agents have also been studied as possible risk factors of lung carcinogenesis. High-risk HPV has drawn a lot of interest because its role as an oncogenic virus is well-established in cervical, anal, and oropharyngeal malignancies [3,5]. Nonetheless, the relationship between HPV and lung cancer remains controversial as a result of its striking geographical heterogeneity and inter-laboratory variability in testing techniques for the presence of virus components (morphology-based or molecular), along with differences in tissue-processing methods [13]. The prevalence of HPV-16 (30.5%) in the present study is higher than the global pooled estimate but is similar to that reported in recent meta-analyses from many Asian countries [10,14-16]. Geographic differences are well established, with HPV prevalence reported at less than 10% in studies from Europe and North America [14], even though higher detection rates have been reported in China [5], Taiwan [6], Japan, and some regions of India [9-16]. Such variation may reflect regional differences in HPV epidemiology, environmental exposures, and genetic susceptibility, as well as potential methodological contributors such as tissue preservation and DNA quality (e.g., PCR primers/target genes). Notably, HPV-18 was absent from all tumor samples. This finding was consistent with previous studies identifying HPV-16 as the most common genotype in malignant pulmonary carcinomas [11,17]. HPV-16 has a stronger oncogenic capacity than HPV-18 for squamous epithelial tissues and is also more frequently linked to extra-genital cancers. However, the absence of HPV-18 in this study should be interpreted with caution, as only two oncogenic genotypes were examined. A broader range of HPV genotyping using next-generation sequencing or multiplex PCR panels could uncover additional oncogenic HPVs implicated in lung carcinogenesis [18].

While adenocarcinoma was the most common histologic type in our cohort, squamous cell carcinoma had a higher proportion of HPV positivity. These data are in concordance with several previous studies [11,19]. Because viral replication is closely linked to epithelial differentiation, the squamous epithelium may be more vulnerable to persistent HPV infection. Histological subtype is important for determining prognosis and guiding subsequent management, as adenocarcinoma is associated with better survival than squamous cell carcinoma [20]. Additionally, HPV-induced dysregulation of the p53 and retinoblastoma pathways can promote malignant transformation in the squamous bronchial epithelium [5,13]. Conversely, a few other investigators have reported similar HPV prevalence across histological subtypes or found no significant association, suggesting that this relation remains unclear [8,21]. Therefore, larger multicenter studies sufficiently powered for histological subtypes are needed.

Smoking is still the most significant established risk factor for lung cancer and was significantly associated with HPV positivity in this study. About two-thirds of HPV-positive patients had tobacco exposure. Cigarette smoke induces chronic epithelial injury, oxidative stress, DNA damage, and impairment of mucociliary clearance, which results in the development of persistent viral infection and local immune suppression [22]. Experimental evidence indicates that tobacco-related mutagens and carcinogens may interact with HPV oncoproteins to increase genomic instability [23] and promote malignant transformation. As a result, HPV infection might act as a cofactor rather than as an independent carcinogenic factor in susceptible hosts. An Iranian case-control study also reported a significant association between smoking and the risk of lung cancer (p < 0.05) [24].

However, the molecular and biological basis for HPV infection-induced lung carcinogenesis is not completely understood. High-risk HPV DNA is integrated into the host genome, resulting in persistent expression of the viral E6 and E7 oncoproteins, which promote p53 degradation and retinoblastoma protein inactivation, thereby stimulating cellular proliferation while inhibiting apoptosis [5-6]. Furthermore, activation of PI3K/Akt [25], EGFR, and Wnt/β-catenin signaling through HPV has also been linked to lung cancer development [13,26]. Chronic inflammation, cytokine dysregulation, and epigenetic changes resulting from persistent viral infection may also promote tumor development [27]. However, detection of HPV DNA alone does not establish active viral oncogenesis. More compelling evidence of biological relevance would include demonstration of viral integration, E6/E7 mRNA expression, or surrogate biomarkers such as p16 overexpression. While molecular studies and immunohistochemical testing have become part of lung cancer diagnosis, the IASLC recommendations still rely on expert consensus rather than mandating them for diagnosis and treatment [28].

Understanding the connection between HPV and lung cancer, in turn, will improve current knowledge of disease pathogenesis and identify potential prognostic or therapeutic responses through biomarker identification. While cancer vaccines have focused largely on host-derived neoantigens and tumor-associated antigens, HPV represents a distinct viral target in a subset of lung cancers. HPV-targeted vaccines remain largely unexplored for lung cancer, but preclinical studies suggest that vaccines targeting HPV E6 and E7 oncoproteins may enhance antitumor immunity in HPV-positive lung tumors [29].

The one epidemiological risk factor showing a constant correlation with lung cancer and mortality is the area of residence. In our study, individuals residing in rural areas demonstrated nearly a threefold higher likelihood of harboring HPV infection as compared to urban residents. This finding implies that socio-geographical circumstances may powerfully shape the influence of HPV in lung carcinoma. Rural populations are frequently exposed to biomass fuel smoke, poorly ventilated cooking environments, recurrent airway irritation, and limited access to preventive and early diagnostic measures [30].

A numerically higher proportion of HPV-positive tumors involved the right lung; however, this association was not statistically significant. This observation has not always been replicated, but anatomical differences in bronchial structure and airflow dynamics have potentially supported a rightward predilection for pulmonary infection or malignancy [31]. However, the present study was not originally designed to test laterality, and this should be regarded with caution.

The present study has several strengths. This study provides data on HPV prevalence in primary lung cancer in eastern India. Histopathological confirmation of all tumors reduced the risk of diagnostic misclassification, and real-time PCR reliably detected HPV DNA in FFPE tissue samples. Additionally, the availability of comprehensive clinicodemographic information enabled evaluation of potential associations between HPV infection and known clinical risk factors.

Despite these strengths, the study has several limitations. First, this study was conducted in a single center with a relatively small sample size, which may limit the generalisability of the findings. Second, for this study, only HPV-16 and HPV-18 were assessed; therefore, we cannot evaluate infections with other oncogenic HPV genotypes. Third, viral DNA was detected by real-time PCR; however, the integration status and E6/E7 transcriptional activity were not determined. Thus, the study cannot establish a causal association of HPV infection with lung carcinogenesis. Fourth, due to the limited follow-up duration and sample size for prognostic factors in this study, long-term survival analyses could not be performed.

Future studies should incorporate multicentre cohorts with larger sample sizes, complete HPV genotyping, quantitative viral load estimation, and measures of oncogene expression. Further clarification of the biological and clinical significance of HPV infection in lung cancer may be achieved by correlating its status with other specific molecular alterations, including amplifications or fusions of EGFR, KRAS, ALK, and ROS1; TP53 mutations; and PD-L1 expression. There is also a need for prospective studies assessing treatment response and survival based on HPV status.

## Conclusions

In this retrospective study of HPV-16 and HPV-18, HPV-16 DNA was detected in nearly one-third of primary lung cancer tissues, whereas HPV-18 was not detected. Smokers, as well as patients with squamous cell carcinoma, were more often HPV positive, and smoking was significantly associated with HPV positivity. Although these observations lend support to the hypothesis that HPV-16 acts as a cofactor in lung carcinogenesis, the findings do not establish a causal relationship. Future multicenter studies that combine detailed HPV genotyping with assessments of viral transcriptional activity, host molecular profiles, and disease outcomes will be needed to clarify the biological and clinical significance of HPV in lung cancer. Further studies are needed to determine whether HPV status has prognostic or therapeutic implications.
